# Annotations

**Published:** 1907-09-21

**Authors:** 


### ANNOTATIONS.

### Medical Inspection of Schools.

As we have always advocated the appointment of a special medical adviser to the Board of Education as a measure essential to secure the efficient hygienic control of the public schools of the country, it is with much satisfaction that we read of the Government's adoption of this proposal. A medical department of the Board is now established as a part of the permanent official organisation, and every confidence, we feel assured, may be entertained in the result. The gentlemen appointed come to their duties with no small store of special experience, and each has a record fully justifying the new responsibilities now committed to his care. Dr. George Newman, in his work as medical officer of health, has proved himself well fitted to deal both with the large problems of hygiene and with the demands of practical organisation. His numerous contributions on various subjects relating to public health have attracted much attention, and his expert knowledge will doubtless now find an appropriate field of application at the Board of Education. Dr. Alfred Eichholz has acted as an inspector under the Board during the last nine years, and has thus gained a thorough knowledge of the questions which must necessarily be considered by the new department; he has also paid special attention to the needs of mentally defective children—an educational problem which cannot be ignored. Much interest and expectation will be aroused by this new development. Without doubt some of the questions involve much difficulty, and will call for delicate handling. Even where the medical position seems manifest, there are social and administrative considerations which involve large matters of principle, and in connection with these controversy is inevitable.

### The Public and Medical Science.

It is a long time since one of the Prime Ministers of the Victorian era proclaimed *sanitas sanitatum omnia sanitas*, to be a sound and wise national policy. The seed has been slow in development, and at times there has seemed reason to doubt whether it might not altogether perish. Yet progress gradually asserts itself, and there are steadily increasing signs that in the mind both of the Government and of the people the care of the public health is becoming recognised as a national claim of the first rank. To the institution of a medical department at the Board of Education we have already referred, and from this much will inevitably arise; it is the beginning, not the end, of large and serious discussions in which public opinion will play a determining part. Having a somewhat similar significance are a number of investigations just sanctioned by the President of the Local Government Board. These include studies of pathogenic bacteria by Dr. Sidney Martin, F.R.S.; bacteriological investigations of the air of sewers and drains by Dr. F. W. Andrewes; observations by Dr. W. G. Savage on the bacteriology of "garget" and maladies of the udder and teats of milch cows, and on the possible relation of these maladies to sore throat in the human subject; and investigations by Dr. H. M. Gordon and Dr. T. J. Horder of the life processes of the meningococcus with a view to the means of combating cerebro-spinal fever. Yet another official nomination may be noted—namely, the appointment by the Royal Commission on Mines of Dr. A. E. Boycott, with a commission to determine whether there are any indications of the existence of ankylostomiasis in the coal miners of Great Britain. In the above brief statement may be found an impressive illustration of the varied interests and penetrating force commanded by medical science, and of the manner in which the public welfare depends on its efficient cultivation and development.

### Medals for Research in Tropical Medicine.

The extraordinary development in the scientific knowledge of tropical diseases which forms so striking a chapter in modern medicine receive a convenient summary in the list of recipients of the Mary Kingsley medal. This medal has been instituted by the Liverpool School of Tropical Medicine to commemorate Miss Mary Kingsley, the African traveller, who died in 1900. The award, which is now announced, confers the medal on the following:—Colonel David Bruce, C.B., F.R.S., who in 1887 discovered the cause of Malta fever and proved that the disease was spread by the milk of infected goats; Dr. A. Laveran, Pasteur Institute, who in 1880 made the discovery that malarial fever is caused by parasites in the blood; Dr. Basile Danilswsky, University of Kharkoff, who discovered numerous parasites in the blood of a large number of animals shortly after Laveran's discovery; Dr. Charles Finlay, chief sanitary officer of Cuba, who in 1880 originated the theory that yellow fever is carried by mosquitoes; Dr. Camirlo Golgi, University of Pavia, who in 1887 made a complete study of the life-cycle of the parasite of malaria; Colonel W. C. Gorgas, United States Army, who as chief sanitary officer of Havana gave practical effect to Finlay's theory, and thus banished yellow fever from the city; W. M. W. Haffkine, C.I.E., whose method of inoculation for plague has been so largely practised in India; Dr. Arthur Loos, Professor of Parasitology, Cairo, for work in connection with parasitology; Dr. Theobald Smith, Harvard University, who in 1893 discovered a new kind of blood parasite in the so-called Texas cattle fever. Professor Robert Koch and Sir Patrick Manson, as was indeed inevitable, also receive the medal, but to refer at length to their scientific achievements would indeed be a work of supererogation. Nevertheless, it is not without interest to note the particular statements which are selected as the official reasons for the presentation of the medal to these gentlemen. Dr. Koch receives credit for having ascertained the cause of cholera, for many other contributions to our knowledge of tropical diseases, and for the discovery of the frequency of malarial infection in children; whilst Sir Patrick Manson is named as the discoverer of the fact that one of the parasites of man belonging to the group of *Filaria* is carried by a species of mosquito. Altogether the list is a notable one, and represents a worthy recognition of true scientific achievement.

